# Exploring Metabolic Interaction between *Ophiostoma novo-ulmi* and *Geosmithia* spp.

**DOI:** 10.1007/s00248-026-02822-2

**Published:** 2026-07-01

**Authors:** Alessia Lucia Pepori, Hari Berto, Alessandra Gionni, Nicola Luchi, Francesco Pecori, Alberto Santini

**Affiliations:** 1 National Research Council – Institute for Sustainable Plant Protection, Via Madonna del Piano 10, Sesto Fiorentino, I-50019 Italy; 2 Department of Agriculture, Food, Environment and Forestry (DAGRI), University of Florence, Firenze, Italy

**Keywords:** *Ophiostoma novo-ulmi*, *Geosmithia* spp., Metabolic interactions, Dutch elm disease, Biolog FF Plates, Carbon substrate utilization, Dual growth rate, Phenotyping

## Abstract

Dutch elm disease (DED), caused by the invasive vascular pathogen *Ophiostoma novo-ulmi*, is one of the most devastating pandemics affecting elms. Within beetle galleries and on elm bark beetle vectors, *O. novo-ulmi* co-occurs with fungi of the genus *Geosmithia*, yet the functional significance of this association remains poorly understood. This study investigates metabolic interactions between *O. novo-ulmi* and *Geosmithia* spp. using in vitro dual-culture experiments and phenotype microarray analysis to elucidate ecological mechanisms potentially influencing disease development and vector ecology. Dual-culture assays on dH_2_O agar revealed that *O. novo-ulmi* subsp. *novo-ulmi* exhibited significantly enhanced radial growth rates when cultured in proximity to *Geosmithia* isolates. Phenotypic analysis revealed distinct metabolic strategies: *O. novo-ulmi* subsp. *novo-ulmi* utilised substrates within 12–24 h and preferred polyols and simple carbohydrates, while *G. pumila* adopted a progressive colonization strategy, ultimately utilising 92 substrates in 90 h and demonstrating greater utilisation of nitrogen-enriched substrates, nucleosides and complex organic acids. Co-culture revealed utilization of adenosine and nitrogen-rich compounds, but antagonistic interactions for polyols and amino sugars. Niche overlap analysis showed asymmetric resource partitioning: *O. novo-ulmi* subsp. *novo-ulmi* utilized 98% of *G. pumila*’s substrates versus 81% reciprocal overlap, while fungal competitiveness analysis indicated *G. pumila* superiority. The distinct metabolic strategies and complementary patterns of substrate utilisation suggest niche construction and the functional partitioning of resources. These findings provide evidence supporting the hypothesis of a functional association within the DED pathosystem. The results highlight the ecological importance of microbial community complexity in pathogenic interactions and suggest that understanding these multitrophic relationships is essential for the development of effective disease management strategies.

**Supplementary Information:**

The online version contains supplementary material available at https://doi.org/10.1007/s00248-026-02822-2.

## Introduction

Since the early 20th century, European elms (*Ulmus* spp.) have been severely threatened by Dutch elm disease (DED), a lethal vascular wilt caused by some invasive fungal pathogens of putative Asian origin belonging to the genus *Ophiostoma* (*Ascomycota*: *Ophiostomatales*,* Ophiostomataceae*), namely *O. ulmi* and *O. novo-ulmi* [1, 2]. The first epidemic wave was triggered by *O. ulmi*, that was later replaced by a more aggressive species, *O. novo-ulmi*, with two subspecies: *O. novo-ulmi* subsp. *novo-ulmi* and subsp. *americana*, which caused a second and more deadly pandemic. The rapid spread of DED is driven by a complex ecological network involving tree hosts, fungal pathogens, bark beetles as vectors (primarily of the genus *Scolytus)*, phoretic mites, virus-like elements, and other associated microorganisms [3, 4]. Recent research has proven the constant presence of fungi belonging to the genus *Geosmithia* (*Ascomycota*: *Hypocreales*, *Bionectriaceae*) within the insect galleries and on insect bodies, where they occupy the same ecosystem, and use similar dispersal mechanisms to those of *O. novo-ulmi* during most of their life cycle [5–9]. Although *Geosmithia* does not exhibit any morphological characteristics that suggest a close association with insects, *Geosmithia* species are closely associated to beetles [9]. Extensive horizontal gene transfer (HGT) of the cerato-ulmin (cu) gene between *O. novo-ulmi* and several *Geosmithia* species has also been found between these two fungi [10]. This genetic exchange reveals a substantially more intricate interaction than simple coexistence; consequently, early studies [5] investigating the nature of this association within the DED pathosystem have proposed a potential hyperparasitic relationship between *Geosmithia* spp. and *O. novo-ulmi*. Further research [7] has substantiated the frequent co-occurrence of *Geosmithia* spp. and *O. novo-ulmi* throughout the DED cycle, while revealing marked variation in their relative abundance according to the epidemiological stage of the disease in natural forest stands.

The ecological distribution of *Geosmithia* species reflects a spectrum of host associations and colonization strategies. While many species function as ecological generalists on hardwood trees, associating opportunistically with multiple bark beetle vectors [9, 11], some have evolved specialized relationships with specific hosts and insect vectors. For instance, *G. morbida* has become functionally dependent on the walnut twig beetle (*Pityophthorus juglandis*) for dispersal and has evolved as a pathogen of black walnut (*Juglans nigra*), causing Thousand Cankers Disease, now spreading across the USA and Europe [12, 13]. Some species have evolved as nutritional ambrosia fungi, establishing obligate mutualistic symbioses with their beetle vectors and colonizing beetle galleries as the primary food source for developing larvae [14]. This functional diversity, ranging from commensal habitat-sharers to specialized nutritional symbionts to pathogens, underscores the ecological complexity of *Geosmithia*-beetle-host plant interactions and supports the hypothesis that the functional role of *Geosmithia* species also within the DED pathosystem extends beyond simple opportunistic co-colonization.

Here we investigated the interactions and metabolic activity of the two fungal genera using in vitro co-culture systems, with the aim of better understanding the mechanisms underlying the complex tri-trophic interactions involving the host (*Ulmus* spp.), the fungal component (*Ophiostoma novo-ulmi* and *Geosmithia* spp.), and its vectors (elm bark beetles). Microbial co-cultures better simulate the complex communities found in nature than monocultures, as microorganisms live in complex microbial communities in the natural environment. Microorganisms interact, share, and exchange metabolic processes and signals, and play a role of synergy or competitive antagonism [15–17]. To this aim, we performed two different experiments: (i) an in vitro dual fungal growth trial onto a minimal substrate was used to assess the effect of the interactions between the two fungal genera in the exploitation of resources and (ii) we used the Biolog system (Biolog Inc., USA) to explore how metabolic-level fungal interactions may influence their ecological roles within the DED pathosystem and how these interactions could impact disease development and potential management strategies. The phenotypic results were also used to test the ability of the two fungi to compete for carbon resources, and the data obtained from the carbon utilization tests enabled the creation of a niche overlap index (NOI) [18] and also the ability to compete with each other .

## Materials and Methods

### Fungal Growth Rate in Dual Culture

Eight *Ophiostoma novo-ulmi* isolates, four of which were *O. novo-ulmi* subsp. *americana* (Onu NAN) and four *O. novo-ulmi* subsp. *novo-ulmi* (Onu EAN), and twelve *Geosmithia* spp. isolates (selected among the most frequently occurring species of *Geosmithia* present on elm trees [5, 6]) were grown in dual culture (Table S1). For each of the 96 fungal combinations, three Petri dishes (90-mm diameter) filled with 25-ml dH_2_O agar (15.0 g bactoagar/ L) substrate were inoculated by placing two 6-mm diameter mycelial plugs (one of *Geosmithia* spp. and one of *Ophiostoma novo-ulmi*), obtained from the edges of actively growing fungal cultures, about 1 cm apart from each other, near to the center of the dish. Cultures were incubated in the dark at 24 °C, and the radii of each colony on the growing edge opposite to the other fungus were measured after 48 h, 3, 5, and 8 days (Fig. S1). Three plates per isolate were inoculated with two identical plugs as a control.

### Fungal Metabolic Potential Analysis

The phenotypic analysis was achieved using OmniLog^®^ ID System multiplate reader (Biolog Inc., Hayward, CA, United States). Phenotype microarray experiments were performed using Biolog FF MicroPlate™ (FF). Each plate contained 95 low-molecular-weight carbon sources from biochemical groups including: amines/amides (*n* = 6), amino acids (*n* = 13), carbohydrates (*n* = 44), carboxylic acids (*n* = 17), polymers (*n* = 5) and miscellaneous/other (*n* = 10) [19]. Each plate also contained water as a negative control (*n* = 1). FF plates were stored at 4 °C until needed. *Geosmithia pumila* (IVV7) and *O. novo-ulmi* subsp. *novo-ulmi* (Onu EAN H328), were selected for this trial since they are the most representative and well-characterized isolates of the species within the DED pathosystem, and have been studied most extensively in recent years [5, 6, 10, 20–26].

Phenotypic analyses were conducted using three inoculation treatments: (i) Onu EAN alone; (ii) *G. pumila* alone; and (iii) a co-inoculation of Onu EAN and *G. pumila.*

Before inoculation, fungi were cultivated on malt extract agar (MEA) for at least one week at 25 °C in the dark to obtain an adequate amount of fungal material from the mycelial growth phase. The superficial layer of the mycelial mat was scraped with a sterile cotton swab to transfer fungal biomass from the colonies (a mixture of spores and mycelial cells) into sterile glass tubes containing 12 ml of an inoculating fluid provided by Biolog. A tissue grinder (Kontes^®^ Duall^®^ 21, Kimble Chase) was used to gently disrupt the fungal biomass to make the inoculum more homogenous. The same suspensions were used to prepare both the single and the co-inoculum. The transmittance of each inoculum was adjusted to 75% (in a Biolog standard turbidimeter) by adding more fungal biomass or inoculation fluid as required. The co-inoculum consisted of a mixture of equal volumes of the single strains’ spore suspensions (20 ml of each inoculum, at 1:1 ratio), which resulted, as well, in a final optical transmission of 75%. Three replica plates were prepared for each of the three analyses: Onu EAN (monoculture), Onu EAN and *G. pumila* (double culture), and *G. pumila* (monoculture). The inoculated plates were kept at 25 °C in darkness in the OmniLog^®^ ID System multiplate reader (Biolog Inc., Hayward, CA, United States). Fungal growth was spectrophotometrically measured as optical density (OD) at 595 nm for 5 days, every 15 min, by the built-in camera and saved as OmniLog units generated by the Biolog^®^ OmniLog software. The OD values of each isolate on the different substrates were normalized against those in the control well [27]. Data analysis for fungal strains’ metabolic profiling was conducted using Kinetic and Parametric software (Biolog). Phenotypes were determined based on the area under the kinetic curve of dye formation [28]. Substrate activation was analyzed by quantifying the number of substrates with metabolic activity at each timepoint up to 90 h. A heatmap dendrogram was produced to show the correlation among the three treatments regarding the utilization of C-sources from the FF plates analyses.

Furthermore, a comprehensive assessment of synergistic substrate utilization, i.e., the extent to which the effect of the two factors together exceeds the effect of teach considered individually, was conducted by measuring the difference between substrate consumption in co-cultures of Onu EAN and *G. pumila* compared to their respective monocultures. Synergy effect was calculated as: synergy effect = ((Onu EAN + *G. pumila*) − (Onu EAN monoculture + *G. pumila* monoculture)/2), as used by [29], where positive values indicate that substrate consumption in co-culture exceeds the sum of individual monocultures (synergistic utilization) and negative values indicate negative interactions. Synergy was analysed as a continuous variable and substrates were grouped into synergistic or antagonistic categories using distribution‑based empirical thresholds [30].

### Statistical Analysis

Daily fungal radial growth rates of each strain of the dual cultures were compared with the respective control cultures by one-way ANOVA (Statistica 10, StatSoft Inc.) with Duncan test (*p* < 0.05).

For the phenotypic data, an OD > 10 threshold was applied, based on preliminary optimization tests, to distinguish true metabolic activity from background signal. An ANOVA was conducted to assess statistical differences for each biochemical group. For groups showing a significant global effect (*p* < 0.05), pairwise comparisons between the three inocula were conducted using Tukey’s Honest Significant Difference (HSD) test. Substrates that yielded a value of zero across all treatments were excluded from the analysis. The statistical analyses were performed on raw data of absorbance values at 90 h. Then, the raw data were normalized to 0–1 range to perform a principal component analysis (PCA). The test was employed to assess the metabolic differentiation between the three inocula. All analysis were performed using R version 4.4.2 [31].

To evaluate the ability of the two fungal species to compete for carbon resources, carbon utilization profiles were used to calculate the Niche Overlap Index (NOI), as well as to assess their competitive interaction. NOI values were calculated according to Lee and Magan [18] and Wilson and Lindow [32]. A NOI value of 0.9 or above indicates a high degree of niche overlap and a competitive disadvantage for the target fungus [18]. We also calculated the ability of Onu EAN and *G. pumila’*s to compete with each other (fungal competitiveness, FC) based on the formula by Blumenstein et al. [33]. If the value of this function is greater than 1.0, one fungus exhibited competitive superiority over the other.

## Results

### Fungal Growth Rate in Dual Culture on Water/Agar

The growth rate of *O. novo-ulmi* strains was generally significantly higher (*p* ≤ 0.05) when paired with *Geosmithia* spp. strains than in monoculture controls (Table 1), despite the assays being conducted on a nutritionally inert substrate (water agar). In particular, Onu EAN (H328) grows significantly more with all *Geosmithia* isolates tested (significant Duncan test, *p* ≤ 0.05). No significant differences in growth rates were observed between *Geosmithia* species when grown with *O. novo-ulmi*, apart from a few isolate-specific interactions, as shown in the table. Within each species, all strains grew at the same rate (non-significant Duncan test, *p* > 0.05).

**Table 1:**
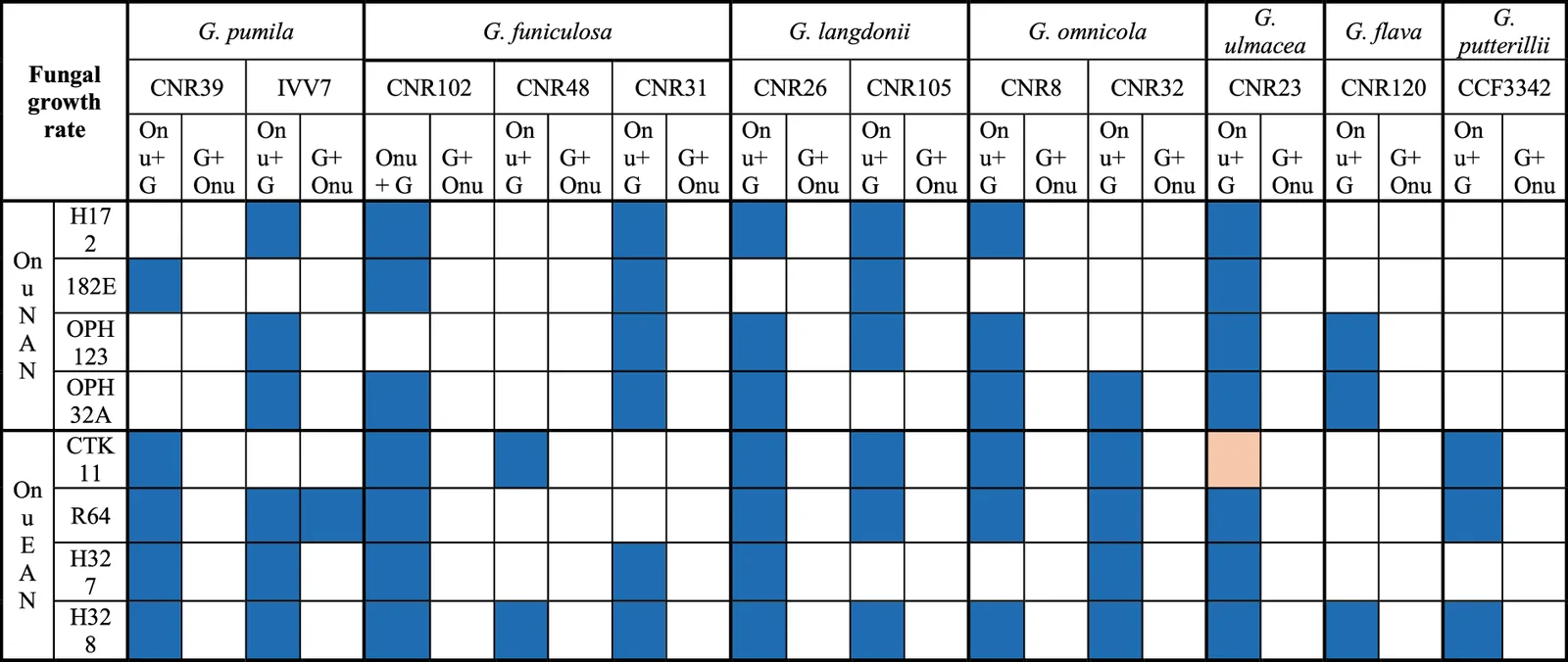
Fungal growth rates in dual culture assays. Blue colour indicates significantly increased growth compared to controls (Duncan’s test, *p* ≤ 0.05); orange colour indicates significantly decreased growth compared to controls (Duncan’s test, *p* ≤ 0.05). *Onu + G* indicates *O. novo-ulmi*’s growth in the presence of *Geosmithia* spp.; *G + Onu* indicates *Geosmithia*’s growth in the presence of *O. novo-ulmi*

### Analysis of Biolog Results

**Fig. 1:**
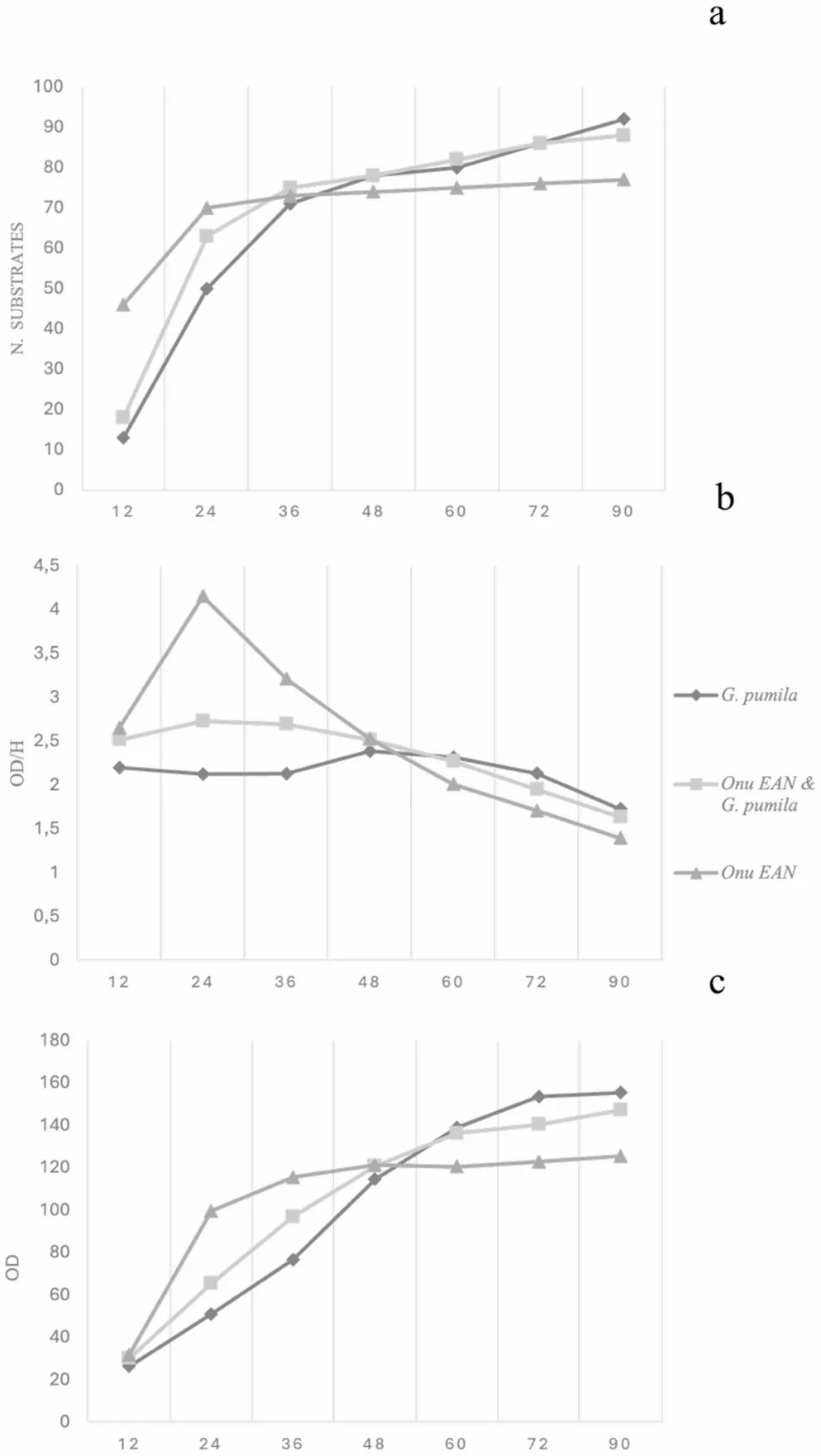
Phenotypic carbon substrate utilization profiles of *Geosmithia pumila* (IVV7), *Ophiostoma novo-ulmi* subsp. *novo-ulmi* (Onu EAN H328), and their co-culture over 90 h. (**a**) The number of substrates that induced metabolic activity, (**b**) hourly substrate consumption rates (OD mean/hours), (**c**) mean OD values indicating total substrate utilization capacity

The three carbon substrate utilization analyses revealed distinct metabolic patterns among the 95 substrates. Three distinct temporal phases characterized the metabolic dynamics of the two fungal species and their co-culture. During the early phase (12–24 h), *G. pumila* initiated substrate utilization slowly, with only 13 substrates at 12 h, progressively increasing to 50 by 24 h. Onu EAN demonstrated a more rapid metabolic onset, utilizing 46 substrates at 12 h and reaching 70 by 24 h. The co-culture of Onu EAN and *G. pumila* displayed intermediate metabolic activity, with 18 substrates at 12 h and 63 substrates by 24 h (Fig. 1a). During the intermediate phase (36–48 h), *G. pumila* accelerated its expansion, while Onu EAN stabilised a plateau. The co-culture matched *G. pumila* performance. During the late phase (60–90 h), *G. pumila* and the co-culture continued its expansion (up to 92 and 88 substrates, respectively), while *O. novo-ulmi* remained stable (77 substrates). Analysis of hourly substrate consumption rates (OD mean/hours) revealed distinct metabolic dynamics among the three treatments. Onu EAN exhibited a pronounced peak of approximately 4.2 at 24 h then decreased rapidly after, indicating intense but transient metabolic activity. In contrast, *G. pumila* and the combined culture maintained relatively stable and had lower consumption rates (2.0–2.5) throughout the observation period. After 24–36 h, all treatments showed a gradual decline in consumption rates, converging toward 1.5–1.8 by 90 h, suggesting a shift from active substrate utilization to a stationary metabolic phase (Fig. 1b). Regarding average carbon substrate utilization, *G. pumila* demonstrated the highest metabolic capacity, achieving approximately 155 OD units by 90 h despite a slower initial growth phase. Conversely, Onu EAN exhibited rapid substrate utilization during the first 48 h but subsequently plateaued at lower levels than the other treatments (Fig. 1c).

**Fig. 2:**
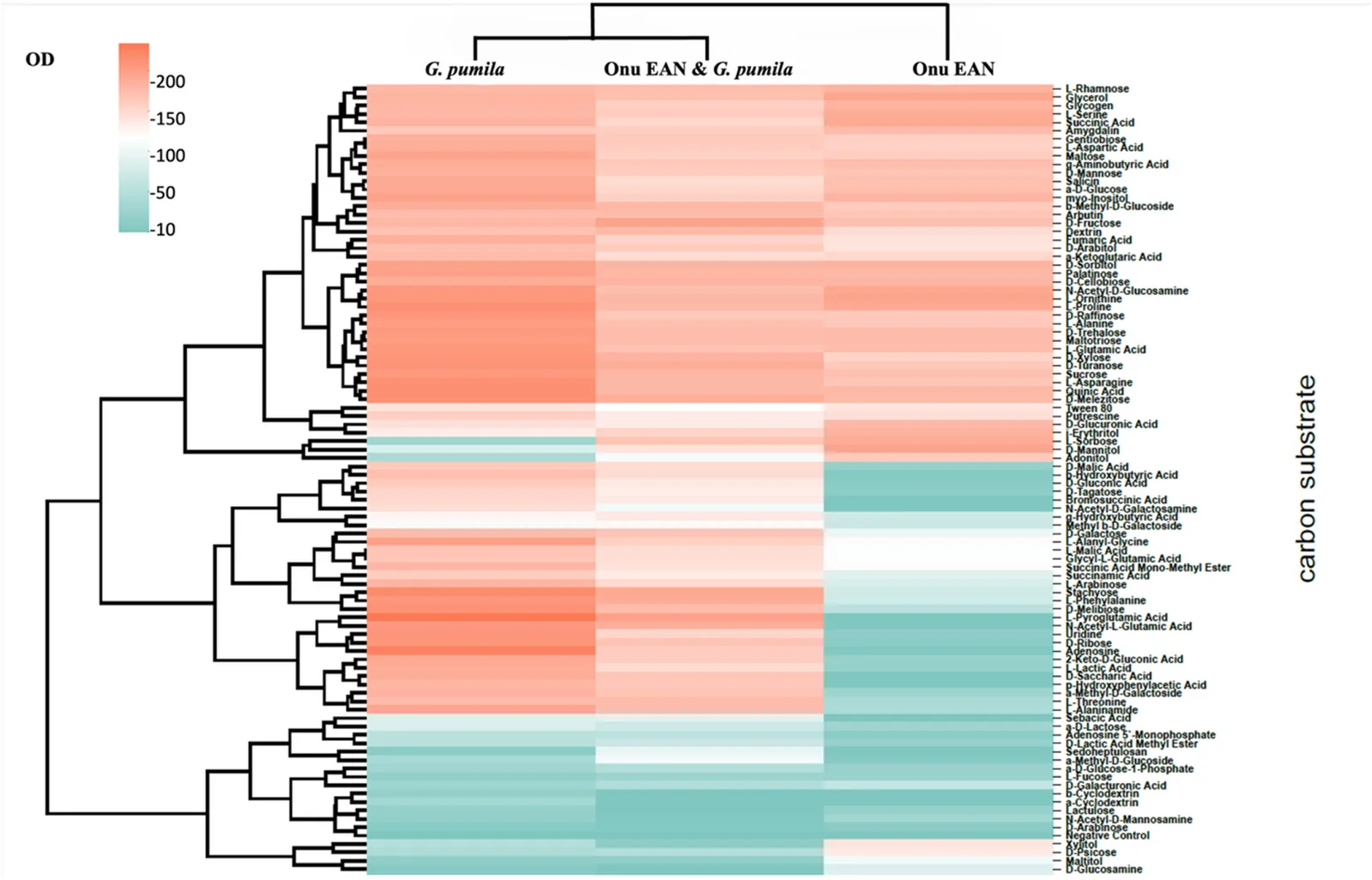
Comparative heatmap of *Geosmithia pumila* (monoculture), *Ophiostoma novo-ulmi* subsp. *novo-ulmi* (Onu EAN) & *G. pumila* (dual culture), and *O. novo-ulmi* subsp. *novo-ulmi* (Onu EAN) (monoculture) strains grown on Biolog FF MicroPlates™ after 90 h based on the utilization of 95 different carbon sources. The green to red color gradient indicates poor to high growth on the respective substrates

Analysis of individual substrate categories revealed that the *G. pumila* strain demonstrated exclusive or superior utilization of complex nitrogen substrates (Fig. 2). N-Acetyl-L-Glutamic Acid, L-Pyroglutamic Acid, N-Acetyl-D-Galactosamine and Sebacic Acid were completely unused by Onu EAN but fully utilized in *G. pumila* treatments and robustly exploited in co-culture (OD 90–165). The use of L-phenylalanine showed a marked rise in co-culture, indicating an increase in the catabolism of aromatic amino acids with greater metabolic diversity. In contrast, the utilization of N-acetyl-D-mannosamine and Lactulose was exploited by both monocultures but not by the co-culture, suggesting direct metabolic antagonism or enzymatic competition.

#### Nucleosides and Nucleotides

Adenosine showed the most pronounced response, remaining nearly unused by Onu EAN in monoculture (OD ~10–25) yet activating to high levels in co-culture (OD 120–195 by 48 h), demonstrating direct metabolic facilitation of the co-culture. Similarly, Uridine showed increased utilization level in co-culture, indicating a *G. pumila* -mediated improvement.

#### Organic Acids

Several carboxylic acids were exclusively or preferentially used by *G. pumila* in monoculture. Bromosuccinic Acid, p-Hydroxyphenylacetic Acid, and D-Saccharic Acid remained unexploited by Onu EAN alone but became metabolically available in dual culture.

#### Polyols and Sugar Alcohols

Onu EAN showed preferential utilization of polyols in monoculture, which were antagonistically suppressed in dual culture. D-Mannitol, L-Sorbose, Xylitol, Adonitol and Maltitol all exhibited marked suppression in co-culture (OD ~ 50–100 vs. 150–200 in Onu EAN monoculture).

#### Monosaccharides

*G. pumila* enhanced the utilization of atypical monosaccharides. D-Ribose, D-Tagatose, D-Gluconic Acid and Stachyose were scarcely used by Onu EAN, while they reached high levels of utilization in dual culture and *G. pumila* monoculture. Conversely, D-Arabinose and L-Fucose remained poorly utilized across all treatments, suggesting limited enzymatic capacity in both species. Sedoheptulosan utilization was absent in Onu EAN (OD = 0) but dramatically increased in co-culture with *G. pumila* (OD = 95.58 vs. 10.39 *G. pumila* monoculture).

#### Oligosaccharides

Cyclodextrin metabolism was *G. pumila* -exclusive: α-Cyclodextrin and β-Cyclodextrin were metabolized just by *G. pumila*, with relatively low OD levels (~ 34–17, respectively).

Comprehensive analysis of 95 distinct carbon substrates revealed substantial heterogeneity in synergistic responses (Table 2). The most pronounced positive synergistic responses were observed for carbohydrate-derived compounds and sugar alcohols. Sedoheptulosan exhibited the strongest synergy (+ 90.39), representing a 90-unit increase in net substrate utilization in the co-culture. Other highly synergistic substrates included α-methyl-D-glucoside (+ 84.50), N-acetyl-L-glutamic acid (+ 84.30), and L-pyroglutamic acid (+ 80.88). Conversely, several substrates exhibited strong negative synergy, indicating competitive inhibition or reduced substrate accessibility under co-culture conditions. Xylitol displayed the strongest antagonistic response (− 82.41), followed by D-glucosamine (− 45.62) and maltitol (− 44.63). Additional substantially antagonistic substrates included D-psicose (− 40.63), succinic acid (− 35.83), and salicin (− 33.26). All these differences were statistically significant (*p* < 0.05).

**Table 2:** Summary of synergistic analysis of 95 substrates comparing the three treatments (*Ophiostoma novo-ulmi* subsp. *novo-ulmi* (Onu EAN) (monoculture), *O. novo-ulmi* subsp. *novo-ulmi* (Onu EAN) and *Geosmithia pumila* (dual culture), and *G. pumila* (monoculture))

	Value
Total substrates analyzed	95
Mean synergy ± SD	9.49 ± 35.96
Synergy range	-82.41 to + 90.39
Positive synergy substrates (n, %)	48 (50.5%)
Negative synergy substrates (n, %)	47 (49.5%)
Mean positive synergy	38.1
Mean negative synergy	-19.74
Strong synergy (> 40) (n, %)	24 (25.3%)
Moderate synergy (10–40) (n, %)	12 (12.6%)
Weak synergy (0–10) (n, %)	11 (11.6%)
Weak antagonism (-10 to 0) (n, %)	12 (12.6%)
Moderate antagonism (10–40) (n, %)	30 (31.6%)
Strong antagonism (<-40) (n, %)	4 (4.2%)

The phenotypic analysis revealed differential utilization of substrate categories among treatments (Table 3). *G. pumila* displayed the highest metabolic activity across most substrates, particularly significantly for amino acids, compared with both treatments. Onu EAN showed lower values, especially significantly lower for carboxylic acids, representing a 62% reduction compared to *G. pumila*. In dual culture, we observed an intermediate profile, with significantly greater use of carboxylic acid than in the Onu EAN monoculture, indicating significant metabolic activation (*p* < 0.05). Polymer degradation remained limited across all treatments with no significant differences (*p* > 0.05)

**Table 3:** Comparison of mean OD values across substrate groups for three treatments (*Ophiostoma novo-ulmi* subsp. *novo-ulmi* (Onu EAN) (monoculture), *Geosmithia. pumila* (monoculture) and *O. novo-ulmi* subsp. *novo-ulmi* (Onu EAN) and *G. pumila* (co-culture)). Values represent the mean ± standard deviation calculated across all substrates within each biochemical group. Different letters indicate significant differences between treatments within the same substrate group (Tukey’s HSD, *p* < 0.05). The number of substrates (n) varies by group. *Glucuronamide* was excluded from the Amines/amides group as it yielded a value of zero across all treatments

	Group	*G. pumila*	Onu EAN	*G. pumila*/ Onu EAN
1	Amines/amides	144^a^±72.97	72^b^±50.52	123^ab^±65.99
2	Amino acids	199^a^±17.92	136^c^±60.95	172^b^±13.21
3	Carbohydrates	143^a^±74.33	119^b^±69.2	136^ab^±59.65
4	Carboxylic acids	163^a^±45.36	68^b^±72.49	144^a^±32.47
5	Miscellaneous	144^a^±68.96	73^b^±79.38	124^a^±45.97
6	Polymers	109^a^±71.95	94^a^±80.71	90^a^±79.3

### PCA Biplot Analysis of Metabolic Differentiation

**Fig. 3:**
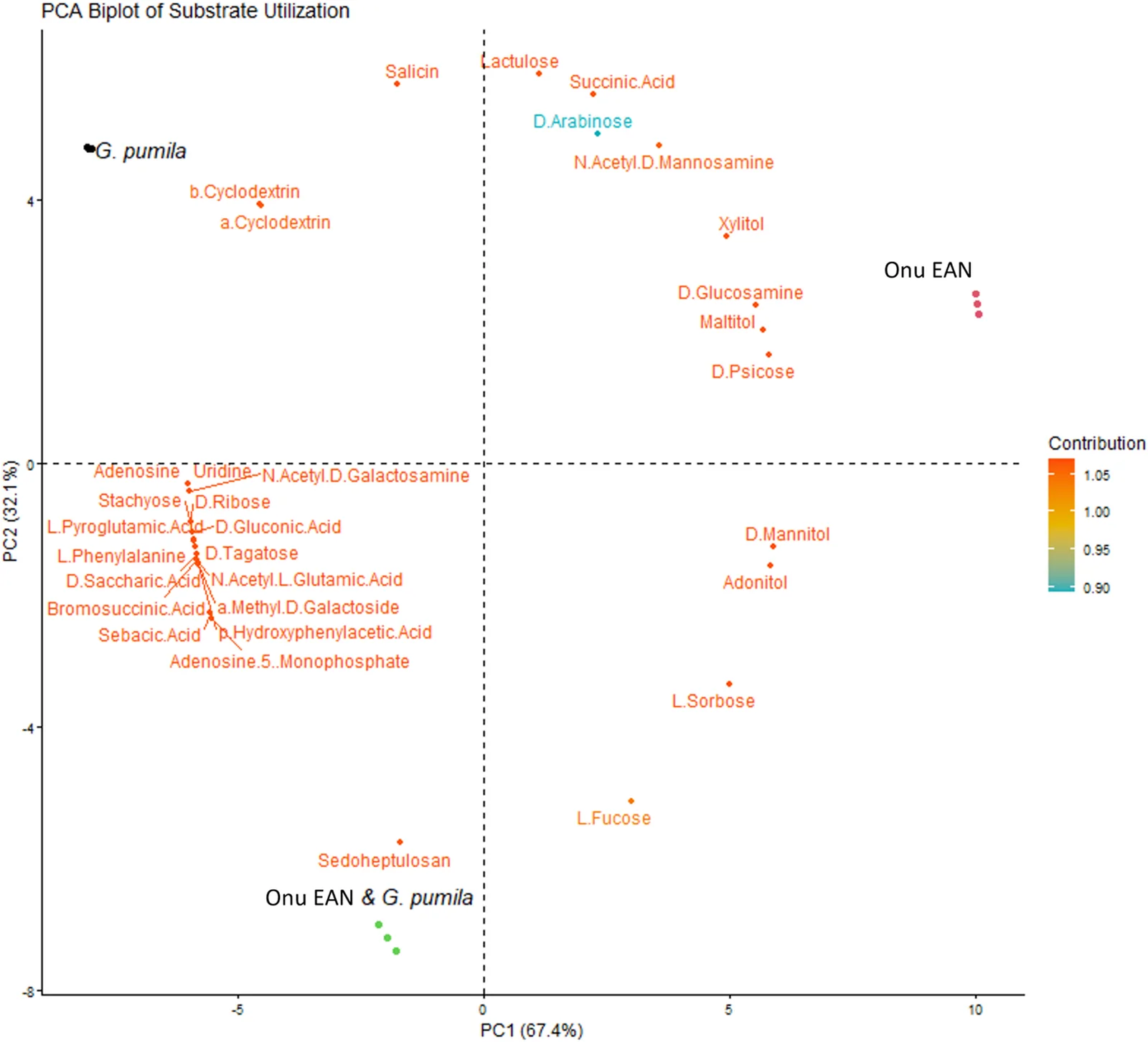
Principal Component Analysis (PCA) biplot demonstrating the metabolic divergence between treatment groups based on 95 carbon substrates. Individual points represent biological replicates (*n* = 3 per treatment) to illustrate within-group variance. PC1 and PC2 together explain 99.5% of the total variance. Vectors (arrows) represent the most relevant contributing substrates, based on major synergic effect; the direction and length of the vectors indicate their influence on the separation of the groups. *Geosmithia pumila* (monoculture black points), *Ophiostoma novo-ulmi* subsp. *novo-ulmi* (Onu EAN) (monoculture pink points) and *O. novo-ulmi* subsp. *novo-ulmi* (Onu EAN) and *G. pumila* (co-culture green points)

Principal component analysis of substrate utilization patterns revealed distinct metabolic separation among Onu EAN *G. pumila*, and their co-culture (Onu EAN and *G. pumila*), with the first two dimensions explaining 99.6% of total variance (PC1: 67.4%, PC2: 32.2%) (Fig. 3). The biplot demonstrated clear spatial clustering of the three treatments, reflecting fundamental differences in substrate preference and enzymatic capacity. Onu EAN occupied the upper-right quadrant, characterised by strong associations with polyols (D-mannitol, xylitol, adonitol, maltitol) and simple carbohydrates. In contrast, *G. pumila* positioned in the upper-left quadrant, showing preferential utilization of nitrogen-enriched substrates (L-pyroglutamic acid, N-acetyl-L-glutamic acid, N-acetyl-D-galactosamine), complex organic acids (bromosuccinic acid, p-hydroxyphenylacetic acid), nucleosides (adenosine, uridine), and exclusive utilisation of cyclodextrins (α and β), that were completely unexploited by Onu EAN. The co-culture occupied the lower left quadrant, reflecting a metabolically integrated system rather than dominance by either partner. Compounds contributing most significantly to PC1 included N-acetyl D-glucosamine, salicin and L-ornithine, indicating carbohydrate and amino acid metabolism as primary discriminatory factors. Compounds clustering along PC2 included various hexoses, pentoses and organic acids, distinguishing strains based on specific metabolic pathways for sugar utilization and organic acid production.

### Ecological Analyses

The analysis of the nutritional profile of the three fungal treatments revealed significant differences in their substrate utilization capacity. Niche overlap index (NOI) analysis revealed moderate nutritional overlap among the strains: Onu EAN shared 98% (66/70) of its substrates with *G. pumila*, while *G. pumila* exhibited 81% (66/85) overlap with Onu EAN’s substrates. These results suggest that the two fungi occupy partially overlapping but distinct nutritional niches.

Regarding fungal competitiveness (FC) according to the Blumenstein et al. [33] model, Onu EAN demonstrated an FC of 0.82 against *G. pumila*, indicating moderate competitive capacity for nutritional resources. In contrast, *G. pumila* showed an FC of 1.21 against Onu EAN, suggesting superior competitiveness in the context of nutritional competition. These findings indicate that, although there is moderate metabolic overlap among the strains, *G. pumila* possesses a greater capacity to compete for shared nutritional substrates.

## Discussion

This study investigated the metabolic and ecological interactions between *Ophiostoma novo-ulmi* and *Geosmithia* spp. within the Dutch elm disease (DED) pathosystem using dual-culture assays and phenotype microarray analyses. *O. novo-ulmi* exhibited a greater capacity for growth on a nutrient-poor medium (water agar). Onu EAN showed an earlier carbon sources utilization, whereas *G. pumila* was capable of exploiting a wider range of carbon sources.

In the culture trial on nutritionally inert water agar substrate, *O. novo-ulmi* showed greater growth when physically close to *G. pumila*, compared to monoculture controls. This is in line with what observed on nutritive media [5], while *Geosmithia* species didn’t show any significant difference from the monoculture.

*Geosmithia* spp. may induce *O. novo-ulmi* faster growth through mechanisms such as modulation of the local microenvironment, or through a biochemical recognition between the two fungi that could cause *O. novo-ulmi* to outcompete *Geosmithia* spp. in terms of spatial occupancy [5]. This is consistent with the current understanding that microbial coexistence often depends on chemical interactions and metabolic plasticity rather than simple competition for resources. This seems to be in line with what was reported by Pepori et al. [5] which observed that *O. novo-ulmi* not only grew faster, but also produced a significantly higher number of perithecia in the presence of *Geosmithia* strains.

The phenotypic analysis provided mechanistic insight into how these metabolic interactions between *G. pumila* and *O. novo-ulmi* may operate. The two extensively characterised fungal isolates [5, 6, 10, 20–26], exhibited distinct metabolic strategies, with Onu EAN demonstrating rapid utilization of a greater number of substrates in the early phase (12–24 h), while *G. pumila* showed colonization on fewer substrates, followed by accelerated expansion until reaching the highest number of activated substrates, at the late stage (60–90 h). This difference in metabolic strategy can be explained by the nature of the fungal pathogens. The pathogenic *O. novo-ulmi* employs a rapid-exploitation strategy on preferred substrates, maximising fitness within a narrow time window, presumably to maintain a high level of pathogenicity towards the host plant. This pattern is also observed in soil pathogens such as *Pythium aphanidermatum* and *Fusarium oxysporum* f.sp. *radicis-lycopersic*i [34]. In contrast, *G. pumila* appears to adopt a more conservative metabolic strategy characterised by a progressive capacity to utilise the substrate throughout the observation period. The co-culture phenotype displayed an intermediate metabolic profile that progressively converges toward the *G. pumila* phenotype. This recalls the well-established principle that metabolically superior competitors, in this case, *G. pumila*, eventually displace the initial colonizers in microbial communities [35].

The synergistic interactions, as the utilization of adenosine, is a widespread phenomenon in microbial communities [17, 36]. Adenosine is a strategic resource that confers metabolic flexibility on the fungus, acting both as a direct source of carbon and nitrogen through its breakdown into ribose and ammonia, and as a host-manipulation effector that triggers the release of otherwise inaccessible nutrients, including glucose [37, 38]. Onu EAN showed minimal adenosine metabolism in monoculture (OD ~ 10–25 at 48 h) but achieved robust utilization in co-culture (OD 120–195 at 48 h), with a 5–19-fold increase. We observed a similar trend for adenosine 5’-monophosphate substrate. This pattern suggests that *G. pumila* enzymatically degrades adenosine into other degradation products accessible to the metabolism of *O. novo-ulmi*, as reported for other fungal interactions [37, 38].

Onu EAN showed a preferential utilization of polyols (D-mannitol, L-sorbose, xylitol, adonitol, maltitol) in monoculture, with OD values ranging from 150 to 200. In dual culture, the utilization of these substrates was significantly suppressed (OD ~ 50–100), suggesting competitive exclusion by *G. pumila*. Similarly, N-acetyl-D-mannosamine was preferentially utilized by Onu EAN alone but antagonistically suppressed in co-culture. Instead, *O. novo-ulmi* likely shifts its metabolic investment towards alternative responses, such as the activation of stress pathways or the mobilisation of internal reserves, independent of external nutrients. This metabolic flexibility is consistent with documented stress responses in pathogenic fungi [39]. These findings reveal an interaction in which *G. pumila* competitively monopolises specific nutritional niches (polyols, certain amino sugars) while simultaneously facilitating *O. novo-ulmi*’s access to alternative and metabolically distinct substrates.

Analysis of the niche overlap index (NOI) quantified this division: Onu EAN exhibited a high overlap with *G. pumila*, shared 98% of its substrates, while the latter showed 81% overlap with Onu EAN substrates, an asymmetry that reflected *Geosmithia*’s broader metabolic capacity. These results, combined with some observed metabolic separation, suggest that the *O. novo-ulmi*-*Geosmithia* system is consistent with niche construction theory, according to which symbiotic partners construct separate niches through their metabolism and activities [40]. In addition, this broader metabolic capacity is consistent with the findings of Pepori et al. [5, 7] who hypothesised that *Geosmithia* sp. is capable of acting as a hyperparasite of *O. novo-ulmi* when other carbon sources are depleted.

Principal component analysis confirmed this niche differentiation, demonstrating a clear metabolic separation (PC1: 67.4%, PC2: 32.2%) in which Onu EAN clustered with metabolic nodes associated with polyols, while *Geosmithia* associated with nitrogen-enriched substrates and nucleosides. Fungal competitiveness analysis has shown that *Geosmithia* utilises shared nutrients more efficiently than Onu EAN, consistent with field observations regarding its presence in competitive habitats [5, 7, 10].

The distinction between metabolic potential, as revealed by phenotypic analysis with defined substrates, and in vitro growth on “poor” substrate is ecologically fundamental to understanding the *O. novo-ulmi*-*Geosmithia* spp. interaction within the DED pathosystem. In naturally colonized host tissues, microhabitat heterogeneity creates zones with variable nutrient availability: fresh xylem tissue provides abundant simple carbohydrates [41] preferred by *O. novo-ulmi* [42], while beetle excrement and degraded tissue within the galleries offer nitrogen-enriched resources [43, 44] exploited by *Geosmithia* spp [45]. It has been observed [45] that the growth of many species of *Geosmithia* is favoured by urea, uric acid, and in part, ammonia, which demonstrates their ability to recycle nitrogen, probably originating from beetle excrement. Since the concentration of nitrogen in beetle galleries greatly affects their vitality [46], the presence of *Geosmithia* spp. for nitrogen recycling seems to be of great importance within the DED pathosystem.

Pathogenic fungi, such as *O. novo-ulmi*, rapidly regulate their metabolism in response to available resources [39]. This metabolic flexibility allows *O. novo-ulmi* to increase overall growth despite limited access to its preferred substrates. This metabolic shift is documented in nutrient-depleted fungal cells as a strategy to sustain essential biosynthetic processes and energy production [47].

The results of this research provide further insights into the interaction between *O. novo-ulmi* and *Geosmithia* spp., offering additional evidence that will help shed light on the true nature of this association in the future [5]. This study suggests that physical contact may establish a finely regulated metabolic exchange and chemical signalling, while the evolutionary stability of horizontal gene transfer events between these fungi (*cerato-ulmin* gene transfer [10]) supports long-term functional integration between these fungal species.

This biological integration, combined with our metabolic data, supports the hypothesis that the two fungi have converged towards a form of functional symbiosis within the DED pathosystem. The distinction between metabolic potential (revealed by phenotypic analysis with microarrays with defined substrates) and growth on nutrient-poor substrates is fundamental from an ecological point of view: in naturally colonized host tissues, microhabitat heterogeneity creates zones with variable nutrient availability, and the ability of fungi to dynamically partition niches, exploit resources at different times, and facilitate growth in the absence of nutrients likely significantly contributes to their ecological success. The metabolic advantages deriving from their interaction are manifold: each fungus seems to facilitate the other’s access to otherwise inaccessible nutrient sources, including through a possible reciprocal exchange of metabolites and enzymatic activities, and *O. novo-ulmi* grows significantly more than in monoculture without affecting *Geosmithia* spp. growth. Collectively, these results suggest that their fungal coexistence may represent a mutualistic strategy conferring selective advantages over solitary survival, thus explaining the evolution of their ecological and biological interaction. Future investigations should focus on deepening our understanding of fungal pathogenesis in the context of complex microbial communities for setting up strategies of disease management in natural ecosystems.

## Supplementary Information

Below is the link to the electronic supplementary material.

Supplementary Material 1

Supplementary Material 2

Supplementary Material 3

## Data Availability

Data will be made available on request.
