# Supplementary material for: Exploring Metabolic Interaction between Ophiostoma novo-ulmi and Geosmithia spp
Source: Microb Ecol. 2026 Jul 1;89:176. doi: 10.1007/s00248-026-02822-2 (PMC13612382; doi:10.1007/s00248-026-02822-2)
Supplement: Supplementary file 1 [file 248_2026_2822_MOESM3_ESM.pdf]

Exploring metabolitic interaction between *Ophiostoma novo-ulmi* and *Geosmithia* spp.

A.L. Pepori[1], H. Berto[1,2], A. Gionni[1], N. Luchi[1], F. Pecori[1], A. Santini[1]

Supplementary material

**Fungal species**	**isolate**	**Geographic orgin**	**Reference**
Ophiostoma novo-ulmi subsp. *americana* (NAN)	H172	Keene, New Hampshire, USA	[1]
	182E	La Favorita (PA), Italy	[2]
	OPH123	Morichella (MC), Italy	CNR IPSP collection
	OPH32A	Monterotondo M.mo (GR), Italy	CNR IPSP collection
Ophiostoma novo-ulmi subsp. *novo-ulmi* (EAN)	CTK11	Chachkam Forest, Iran	[2]
	R64	Hermeus , Romania	[2]
	H327	Brezno-Nizke, Tatry, Bratislava, Slovakia	[1]
	H328	Gottschalf Charkov, Russia	[3]
Geosmithia pumila	CNR39	Vilegia, Central Bohemia, Czech Rep.	[4]
	IVV7	Vibo Valentia (RC), Italy	[5]
G. funiculosa	CNR102	Libis, Central Bohemia, Czech Rep.	[4]
	CNR48	Vilegia, Central Bohemia, Czech Rep.	[4]
	CNR31	Vilegia, Central Bohemia, Czech Rep.	[4]
G. langdonii	CNR105	Hostenice, Usti nad Labem, Czech Rep.	[4]
	CNR26	Vilegia, Central Bohemia, Czech Rep.	[4]
G. omnicola	CNR8	Vilegia, Central Bohemia, Czech Rep.	[4]
	CNR32	Stredokluky, Central Bohemia, Czech Rep.	[4]
G. ulmacea	CNR23	Stredokluky, Central Bohemia, Czech Rep.	[4]
G. flava	CNR120	Marsovice, Czech Rep.	[4]
G. putterillii	CCF3342	North Bohemia, Louny, Czech Rep.	[6]

Table S1. *Ophiostoma novo ulmi* and *Geosmithia* spp. fungi strains used in this work.

A **B**

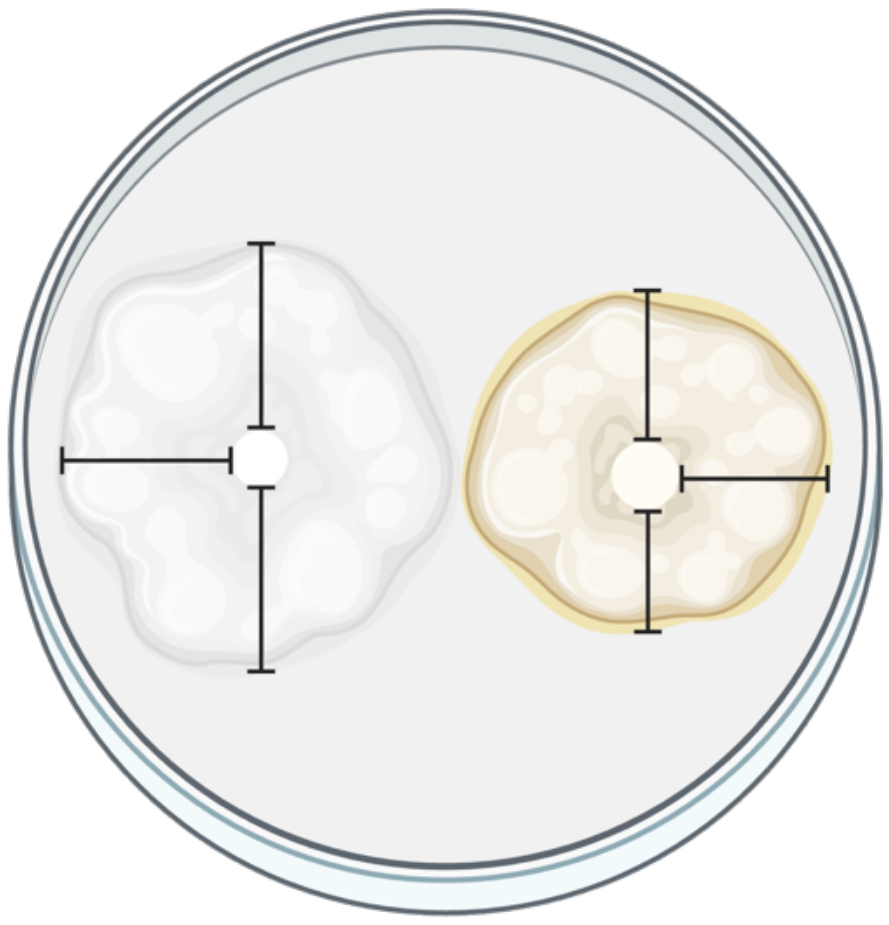

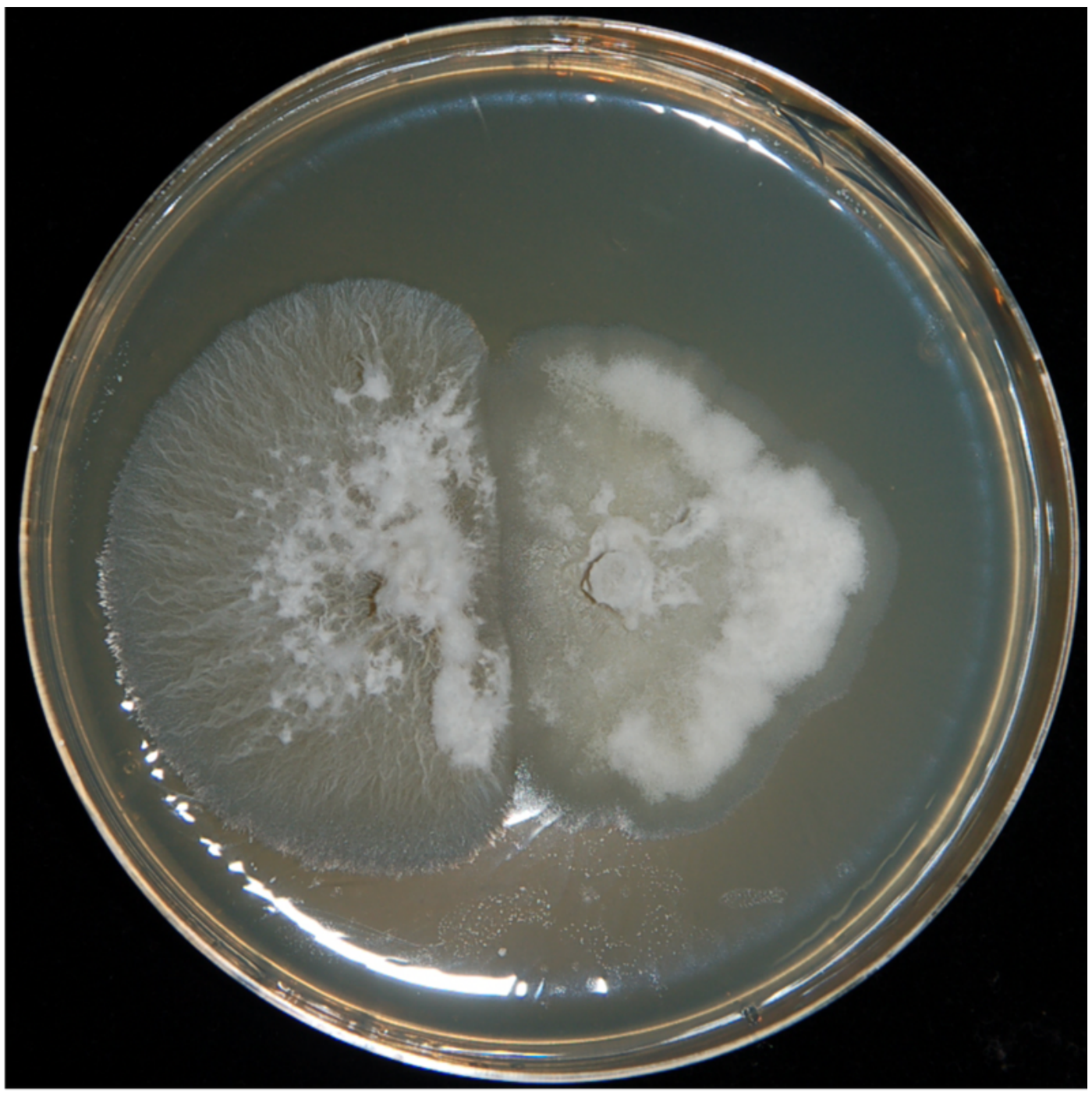

Figure S1. Fungal Growth Rate in Dual Culture. (A) Schematic diagram illustrating the cross-sectional measurement method used to measure the radial mycelial growth increments: two fungal colonies are inoculated on the same agar plate, and growth is recorded along perpendicular axes from the inoculation point. (B) Photo of a representative Petri dish showing two fungal colonies grown on water/agar medium, *Ophiostoma novo-ulmi* (right) and *Geosmithia* spp. (left).

Reference

1. Pipe N, Buck K, Brasier C (1995) Genomic fingerprinting supports the separation of Ophiostoma piceae into two species. Mycological Research 99:1182–1186

2. Santini A, Fagnani A, Ferrini F, et al (2005) Variation among Italian and French elm clones in their response to *Ophiostoma novo-ulmi* inoculation. Forest Pathology 35:183–193. https://doi.org/10.1111/j.1439-0329.2005.00401.x

3. Brasier CM (1986) Comparison of pathogencity and cultural characteristics in the EAN and NAN aggressive subgroups of *Ophiostoma ulmi*. Transactions of the British Mycological Society 87:1–13. https://doi.org/10.1016/S0007-1536(86)80001-8

4. Pepori AL, Kolařík M, Bettini PP, et al (2015) Morphological and molecular characterisation of *Geosmithia* species on European elms. Fungal Biology 119:1063–1074. https://doi.org/10.1016/j.funbio.2015.08.003

5. Scala A, Comparini C, Tegli S, Scala F (2007) A non-*Ophiostoma* fungus expresses the gene encoding the hydrophobin cerato-ulmin. Journal of Plant Pathology 233–240

6. Kolarik M, Kubátová A, Pažoutová S, others (2004) Morphological and molecular characterisation of *Geosmithia putterillii* , *G. pallida* comb. nov. and *G. flava* sp. nov., associated with subcorticolous insects. Mycological research 108:1053–1069
